# Associations between Environmental Quality and Mortality in the Contiguous United States, 2000–2005

**DOI:** 10.1289/EHP119

**Published:** 2016-10-07

**Authors:** Yun Jian, Lynne C. Messer, Jyotsna S. Jagai, Kristen M. Rappazzo, Christine L. Gray, Shannon C. Grabich, Danelle T. Lobdell

**Affiliations:** 1 Oak Ridge Institute for Science and Education, National Health and Environmental Effects Research Laboratory (NHEERL), U.S. Environmental Protection Agency (EPA), Chapel Hill, North Carolina, USA; 2 School of Community Health, College of Urban and Public Affairs, Portland State University, Portland, Oregon, USA; 3 Division of Environmental and Occupational Health Sciences, School of Public Health, University of Illinois, Chicago, Chicago, Illinois, USA; 4 NHEERL, U.S. EPA, Chapel Hill, North Carolina, USA; 5 UNC Gillings School of Global Public Health, Chapel Hill, North Carolina, USA

## Abstract

**Background:**

Assessing cumulative effects of the multiple environmental factors influencing mortality remains a challenging task.

**Objectives:**

This study aimed to examine the associations between cumulative environmental quality and all-cause and leading cause-specific (heart disease, cancer, and stroke) mortality rates.

**Methods:**

We used the overall Environmental Quality Index (EQI) and its five domain indices (air, water, land, built, and sociodemographic) to represent environmental exposure. Associations between the EQI and mortality rates (CDC WONDER) for counties in the contiguous United States (*n* = 3,109) were investigated using multiple linear regression models and random intercept and random slope hierarchical models. Urbanicity, climate, and a combination of the two were used to explore the spatial patterns in the associations.

**Results:**

We found 1 standard deviation increase in the overall EQI (worse environment) was associated with a mean 3.22% (95% CI: 2.80%, 3.64%) increase in all-cause mortality, a 0.54% (95% CI: –0.17%, 1.25%) increase in heart disease mortality, a 2.71% (95% CI: 2.21%, 3.22%) increase in cancer mortality, and a 2.25% (95% CI: 1.11%, 3.39%) increase in stroke mortality. Among the environmental domains, the associations ranged from –1.27% (95% CI: –1.70%, –0.84%) to 3.37% (95% CI: 2.90%, 3.84%) for all-cause mortality, –2.62% (95% CI: –3.52%, –1.73%) to 4.50% (95% CI: 3.73%, 5.27%) for heart disease mortality, –0.88% (95% CI: –2.12%, 0.36%) to 3.72% (95% CI: 2.38%, 5.06%) for stroke mortality, and –0.68% (95% CI: –1.19%, –0.18%) to 3.01% (95% CI: 2.46%, 3.56%) for cancer mortality. Air had the largest associations with all-cause, heart disease, and cancer mortality, whereas the sociodemographic index had the largest association with stroke mortality. Across the urbanicity gradient, no consistent trend was found. Across climate regions, the associations ranged from 2.29% (95% CI: 1.87%, 2.72%) to 5.30% (95% CI: 4.30%, 6.30%) for overall EQI, and larger associations were generally found in dry areas for both overall EQI and domain indices.

**Conclusions:**

These results suggest that poor environmental quality, particularly poor air quality, was associated with increased mortality and that associations vary by urbanicity and climate region.

**Citation:**

Jian Y, Messer LC, Jagai JS, Rappazzo KM, Gray CL, Grabich SC, Lobdell DT. 2017. Associations between environmental quality and mortality in the contiguous United States, 2000–2005. Environ Health Perspect 125:355–362; http://dx.doi.org/10.1289/EHP119

## Introduction

Many environmental factors influence mortality. The majority of studies investigating environmental effects on mortality have focused on air pollutants and show that exposure to air pollutants is linked with an increased risk of mortality (e.g., Krall et al. 2013; Tao et al. 2012; Zanobetti and Schwartz 2009). Associations between water pollutants and mortality have also been reported, particularly in the vicinity of polluted water environments (Harmon and Coe 1993; Hendryx et al. 2012; Ren et al. 2015). A few studies have also reported associations between mortality and other aspects of environmental quality. Elevated levels of underground radon were found to be linked with an increased risk of mortality due to lung cancer and chronic obstructive pulmonary disease (Turner et al. 2012a, 2012b). Higher cancer mortality was found in the vicinity of incinerators and hazardous waste sites (García-Pérez et al. 2013). Additionally, disparities in mortality rates have been observed across sociodemographic environments such as income, education, and immigration (Ezzati et al. 2008; Pearce et al. 2010, 2011). Hence, environmental conditions are clearly important factors that affect human mortality.

The influences of different environmental factors often occur in tandem; however, few studies have explored the impact on mortality of multiple exposures across environmental domains (Pearce et al. 2010, 2011). Most mortality studies have focused on the independent effect of a single variable (e.g., the association between one air pollutant and mortality) (Lu et al. 2015; Schwartz et al. 2015) or the combined effects of a few (typically two to three) variables within the same environmental domain (e.g., fine and coarse particulate matter in the air) (Tao et al. 2012; Zanobetti and Schwartz 2009). A few studies have assessed the overall impact of water environment on mortality (Hendryx et al. 2012; Ren et al. 2015). Although some work has been performed outside the United States (Pearce et al. 2010, 2011), the relationships between mutiple environmental factors and mortality in the United States remain under-explored.

Mortality has also been observed to vary spatially, and some of that spatial variability occurs across rural–urban differences. Higher mortality has been observed in nonmetropolitan than in metropolitan areas (Cossman et al. 2010). However, the underlying causes of this difference remain unclear. It seems likely that urban and rural residents are exposed to different environments; for example, urban residents may experience higher amounts of air pollution, whereas rural residents may be exposed to more agricultural pesticide usage. The health impacts of environmental quality may both result from and differ across the rural–urban continuum. However, most studies about environmental impact on mortality have only focused on urban areas (Krall et al. 2013; Zanobetti and Schwartz 2009). Therefore, analyses exploring the differences in environmental impacts on mortality across urbanicity are needed.

The associations between cumulative environmental quality and mortality may also be influenced by climate. Previously, temperature was found to modify the association between air pollution and mortality (Kim et al. 2015). Associations between particulate matter and mortality also differed across climate regions (Zanobetti and Schwartz 2009). In regard to climate and sociodemographic environments, persons with lower income showed reduced resistance to extreme weather conditions such as heat/cold waves and natural disasters (Nordio et al. 2015; O’Neill et al. 2005). However, studies on spatial variations in environmental impacts on mortality across different climates are rare, and efforts are needed to target places with high environmental burdens on mortality.

To address these gaps, we used the Environmental Quality Index (EQI) to assess the cumulative environmental effect on mortality and the spatial patterns of that effect. The EQI was constructed to represent county-level ambient environmental quality across the United States (Lobdell et al. 2011; Messer et al. 2014); it encompasses an overall index and five domain indices (air, water, land, built, and sociodemographic). In the present study, we investigated the associations between the overall EQI and all-cause and cause-specific mortality rates for the contiguous United States. We further examined the associations for the EQI domain indices to assess different effects across different environmental aspects. Finally, we investigated spatial patterns of associations by urbanicity and climate.

## Methods

### Mortality Outcome Data

Age-adjusted mortality rates for the contiguous United States in 2000–2005 were obtained from the Wide-ranging Online Data for Epidemiologic Research system of the U.S. Centers for Disease Control and Prevention (CDC) (2015). Age-adjusted mortality rates were weighted averages of the age-specific death rates, and they were used to account for different age structures among populations (Curtin and Klein 1995). The mortality rates for counties with < 10 deaths were suppressed by the CDC to protect privacy and to ensure data reliability; only counties with ≥ 10 deaths were included in the analyses. The underlying cause of mortality was specified using the World Health Organization’s *International Statistical Classification of Diseases and Related Health Problems* (10th revision; ICD-10). In this study, we focused on the all-cause mortality rate (A00-R99) and on mortality rates from the three leading causes: heart disease (I00-I09, I11, I13, and I20-I51), cancer (C00-C97), and stroke (I60-I69) (Heron 2013). We excluded mortality due to external causes for all-cause mortality, as has been done in many previous studies (e.g., Pearce et al. 2010, 2011; Zanobetti and Schwartz 2009), because external causes of mortality are less likely to be related to environmental quality. We also focused on the contiguous United States because the numbers of counties with available cause-specific mortality rates were small in Hawaii and Alaska. County-level rates were available for 3,101 of the 3,109 counties in the contiguous United States (99.7%) for all-cause mortality; for 3,067 (98.6%) counties for heart disease mortality; for 3,057 (98.3%) counties for cancer mortality; and for 2,847 (91.6%) counties for stroke mortality.

### Environmental Quality Index

**Figure 1:**
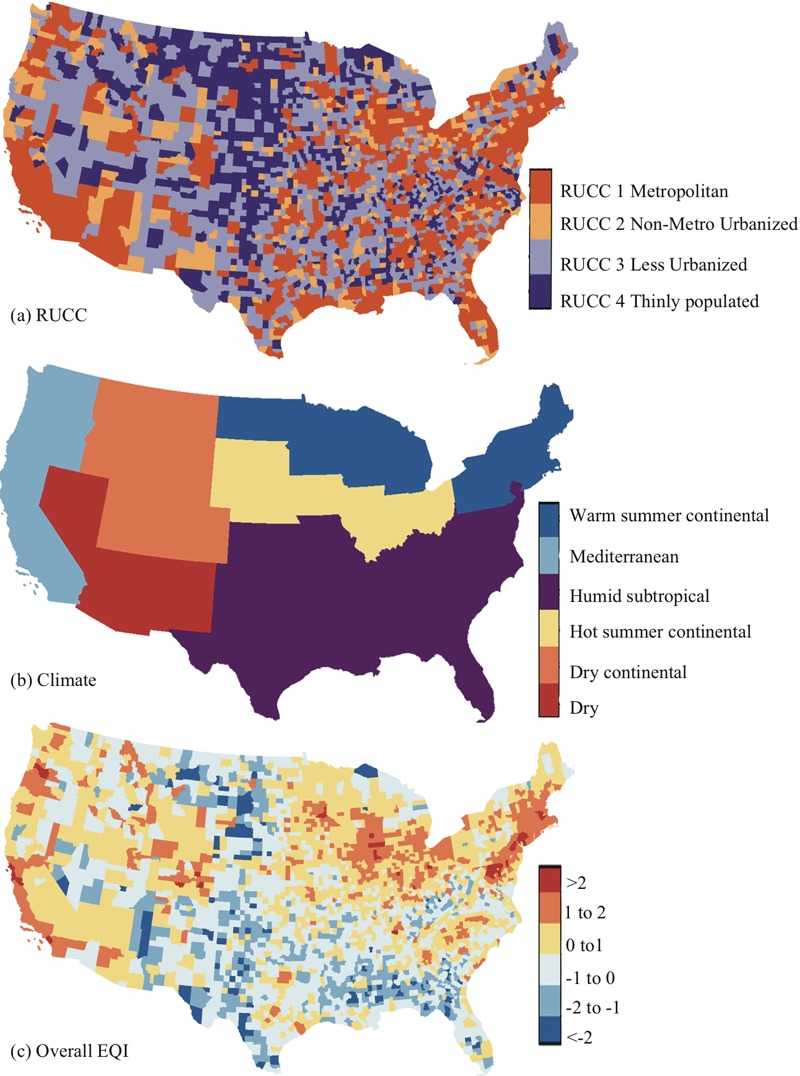
Maps for (*A*) the condensed Rural–Urban Contimuum Code RUCC, (*B*) condensed climate regions, and (*C*) rescaled overall Environmental Quality Index (EQI) (mean = 0 and standard deviation = 1) in the contiguous United States. Maps were downloaded from the U.S. Census Bureau (2010; https://www.census.gov/geo/maps-data/data/tiger.html) and were reproduced using R (version 3.0.3; R Project for Statistical Computing).

The Environmental Quality Index (EQI) was used to represent cumulative environmental quality for 2000–2005 (Figure 1). Methods for calculating the EQI have been published elsewhere (Lobdell et al. 2011; Messer et al. 2014). Briefly, the EQI was constructed using principal component analysis (PCA) to reduce 219 environmental variables into five domain-specific (air, water, land, built, and sociodemographic) indices. These indices were then included in a second PCA to produce an overall environmental quality index. The 18 data sources for the EQI ranged from the Air Quality System in the air domain to Uniform Crime Reports in the sociodemographic domain; a complete list has been published by Lobdell et al. (2011) and the U.S. Environmental Protection Agency (EPA) (2014). The original EQI covered the entire United States at the county level. The summary statistics of the EQI for the contiguous United States are listed in Table S1. Higher EQI values represent worse environmental quality. The EQI was constructed for the entire 6-year period of 2000–2005; because of data unavailability (infrequency of update), single-year EQIs were not constructed (Messer et al. 2014). Overall and domain-specific EQI data were downloaded from the U.S. EPA (2015).

### Covariates

We used county-level Rural–Urban Continuum Codes (RUCCs) to account for variability across urbanicity [U.S. Department of Agriculture (USDA) 2015]. The 9 RUCC groups were condensed into 4 groups, as has been done elsewhere: metropolitan urbanized (RUCC1), nonmetropolitan urbanized (RUCC2), less urbanized (RUCC3), and thinly populated (RUCC4) areas (Langlois et al. 2010; Luben et al. 2009; Messer et al. 2010) (Figure 1). The exclusion of counties with < 10 deaths had the largest impact on stroke mortality in thinly populated areas (RUCC4), with 217 counties removed (~20% of the counties in this category); for other types of mortality or other RUCCs, < 50 counties were excluded. We also used Köppen climate regions to assess potential variability under different climates (Kottek et al. 2006). Originally, counties were classified into 18 climate regions in the contiguous United States according to their annual and monthly averages of temperature and precipitation. Here, the 18 climate regions were condensed into 6 groups including dry, dry continental, hot summer continental, humid subtropical, Mediterranean, and warm summer continental regions, as in a previous study (Figure 1; see also Table S2) (Zanobetti and Schwartz 2009). The exclusion of counties with < 10 deaths had the largest impact on stroke mortality in the dry continental region, with 68 counties removed (~20%). We further constructed a variable combining the RUCC and climate region categories to explore the variation in the associations linked with both urbanicity and climate (24 groups). This variable represented unique RUCCs in each climate region: for example, metropolitan urbanized areas in humid subtropical regions. The RUCCs, the climate regions, and combinations of the two were included as regional cluster variables (random effects) in hierarchical models. The number of counties in each RUCC, in each climate region, and each combination can be found in Table 1 (see also Table S3). Other county-level sociodemographic variables (not used in the sociodemographic index) adjusted in the analysis included the percent of white population and the population density (U.S. Census Bureau 2010, 2012). Estimated county-level cigarette smoking from Dwyer-Lindgren et al. (2014) and alcohol consumption from Dwyer-Lindgren et al. (2015) were also used for adjustment. These data were available for all counties in the United States.

**Table 1:** Mean (95% CI) age-adjusted death rates per 1,000 population and number of counties (*n*) in RUCCs and climate regions in the contiguous United States (2000–2005).

RUCC/Climate	All-cause		Heart disease		Stroke		Cancer	
	Mean (95% CI)	*n*	Mean (95% CI)	*n*	Mean (95% CI)	*n*	Mean (95% CI)	*n*
RUCC1 Metropolitan urbanized	8.18 (6.06, 10.30)	1,085	2.42 (1.50, 3.34)	1,084	0.59 (0.34, 0.84)	1,069	1.98 (1.51, 2.45)	1,085
RUCC2 Nonmetro urbanized	8.28 (6.26, 10.30)	319	2.48 (1.52, 3.44)	319	0.60 (0.33, 0.87)	319	1.98 (1.55, 2.41)	319
RUCC3 Less urbanized	8.4 (5.91, 10.89)	1,049	2.59 (1.45, 3.73)	1,049	0.62 (0.29, 0.95)	1,020	1.99 (1.44, 2.54)	1,048
RUCC4 Thinly populated	7.8 (5.00, 10.60)	648	2.43 (1.27, 3.59)	615	0.61 (0.34, 0.88)	439	1.92 (1.27, 2.57)	605
Dry climate	7.54 (5.31, 9.77)	65	2.16 (1.24, 3.08)	63	0.48 (0.28, 0.68)	52	1.75 (1.18, 2.32)	62
Dry continental climate	7.17 (5.05, 9.29)	212	1.96 (1.25, 2.67)	204	0.56 (0.32, 0.80)	147	1.72 (1.13, 2.31)	202
Hot summer continental climate	7.73 (5.57, 9.89)	538	2.35 (1.53, 3.17)	524	0.58 (0.34, 0.82)	490	1.93 (1.46, 2.40)	523
Humid subtropical climate	8.77 (6.59, 10.95)	1,662	2.72 (1.70, 3.74)	1,656	0.65 (0.34, 0.96)	1,569	2.06 (1.53, 2.59)	1,649
Mediterranean climate	7.39 (5.90, 8.88)	133	2.02 (1.39, 2.65)	132	0.60 (0.44, 0.76)	124	1.86 (1.49, 2.23)	132
Warm sumer continental climate	7.44 (5.83, 9.05)	491	2.23 (1.43, 3.03)	488	0.52 (0.32, 0.72)	465	1.89 (1.52, 2.26)	489
Notes: CI, confidence interval; RUCC: Rural–Urban Continuum Code.								

### Statistical Analysis

We assessed the relationships between county-level exposures and mortality rates. We analyzed the associations between overall EQI and mortality and between EQI domain indices and mortality. For both overall EQI and domain indices, we performed the following analyses: *a*) a multiple linear regression model to assess the average effects for the contiguous United States; *b*) a random intercept, random slope hierarchical model clustered by the condensed RUCC categories to assess variations in associations by urbanicity; *c*) a random intercept, random slope hierarchical model clustered by the condensed climate regions to assess variations linked with climate; and *d*) a random intercept, random slope hierarchical model clustered by the RUCC–climate combinations to assess variations linked with both urbanicity and climate. Models were built separately for all-cause and cause-specific mortality rates. For domain-specific models, all indices were included simultaneously. All of the mortality rates in the model were natural log–transformed, and the EQI indices were rescaled (mean = 0, standard deviation = 1) to facilitate model convergence and interpretation. Following these transformations, the results were reported as percent difference in mortality rate and 95% confidence interval (95% CI) per 1 standard deviation (SD) increase in the overall EQI or domain-specific EQI indices. All of the analyses were performed in R 3.2.0 (R Project for Statistical Computing) with the package “lme4” (Bates et al. 2015).

An example of the overall EQI model clustered by RUCC for all-cause mortality was


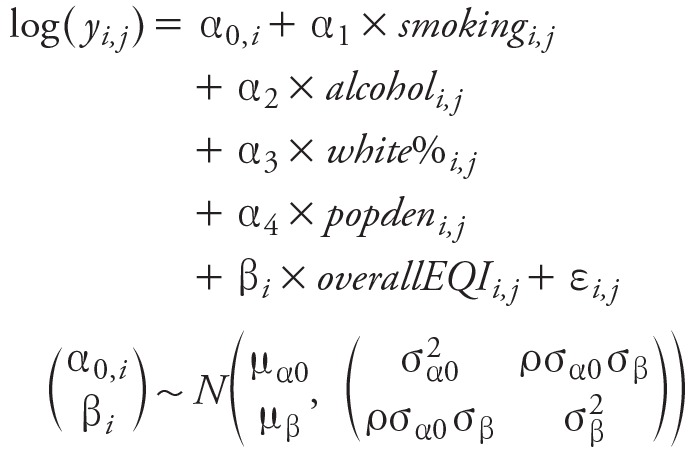


Where *y* was the county-level age-adjusted all-cause death rate; *smoking* was the county-level cigarette smoking prevalence; *alcohol* was the county-level drinking prevalence; *white%* was the percent of the white population; *popden* was population density; *overallEQI* was the overall EQI index; *i* was the index for cluster (RUCCs in this model); *j* was the index for county; α_(0,_ *_i_*_)_, α_1_–α_4_ and β*_i_* were model coefficients. We further assumed the coefficients α_(0,_ *_i_*_)_ and β*_i_* were from a bivariate normal distribution where μ_α0_ and μ_β_ were the expectations, σ^^2^^__α0__ and σ^^2^^__β__ were the variances for α_(0,_ *_i_*_)_ and β*i* respectively, and ρ was the correlation coefficient.

## Results

### Population Description

The average annual county-level age-adjusted all-cause mortality rate was 8.19 deaths (95% CI: 5.78, 10.60) per 1,000 population. The mortality rates were 2.49 (95% CI: 1.43, 3.55) for heart disease mortality, 1.97 (95% CI: 1.44, 2.50) for cancer mortality, and 0.61 (95% CI: 0.32, 0.90) for stroke mortality. The mortality rates varied across RUCCs and climate regions (Table 1). In general, RUCC-stratified mortality rates were highest in less-urbanized areas (RUCC3) and lowest in thinly populated areas (RUCC4). Among climate regions, mortality rates were highest in the humid subtropical region and lowest in the dry continental region.

### Associations between EQI and Mortality for the Contiguous United States

We found positive associations between the overall EQI and mortality for the contiguous United States, suggesting that worse cumulative environmental quality was associated with higher mortality rates. The multiple linear regression models showed that a 1-SD increase in the overall EQI was associated with a 3.22% (95% CI: 2.80%, 3.64%) increase in the all-cause mortality rate, a 0.54% (95% CI: –0.17%, 1.25%) increase in the heart disease mortality rate, a 2.71% (95% CI: 2.21%, 3.22%) increase in the cancer mortality rate, and a 2.25% (95% CI: 1.11%, 3.39%) increase in stroke mortality.

The associations varied for EQI domains, and they ranged from –1.27% (95% CI: –1.70%, –0.84%) to 3.37% (95% CI: 2.90%, 3.84%) for all-cause mortality, –2.62% (95% CI: –3.52%, –1.73%) to 4.50% (95% CI: 3.73%, 5.27%) for heart disease mortality, –0.88% (95% CI: 2.12%, 0.36%) to 3.72% (95% CI: 2.38%, 5.06%) for stroke mortality, and –0.68% (95% CI: –1.19%, –0.18%) to 3.01% (95% CI: 2.46%, 3.56%) for cancer mortality (Table 2). Air had the largest associations with all-cause, heart disease, and cancer mortality, whereas the sociodemographic index had the largest association with stroke mortality.

**Table 2:** Percent difference (95% CI) in mortality rates per 1-standard-deviation increase in EQI domain indices from the multiple linear model.

EQI domains	All-cause	Heart disease	Stroke	Cancer
Air	3.37 (2.90, 3.84)	4.50 (3.73, 5.27)	–0.88 (–2.12, 0.36)	3.01 (2.46, 3.56)
Water	–0.26 (–0.59, 0.08)	–1.70 (–2.25, –1.15)	0.50 (–0.32, 1.31)	–0.56 (–0.95, –0.17)
Land	–1.27 (–1.70, –0.84)	–1.81 (–2.51, –1.11)	–0.65 (–1.69, 0.39)	–0.68 (–1.19, –0.18)
Built	1.19 (0.77, 1.60)	–0.77 (–1.48, –0.05)	1.04 (–0.12, 2.20)	0.09 (–0.43, 0.61)
Sociodemographic	–0.31 (–0.86, 0.24)	–2.62 (–3.52, –1.73)	3.72 (2.38, 5.06)	0.60 (–0.05, 1.24)
Notes: CI, confidence interval; EQI, Environmental Quality Index.				

### Associations between EQI and Mortality Clustered by RUCC

The associations for hierarchical models clustered only by RUCC were positive for all-cause, stroke, and cancer mortality for all RUCC groups (Table 3). The associations for heart disease mortality were positive for metropolitan urbanized areas (RUCC1) and negative for other RUCC groups. The ranges of percent difference in mortality rates per 1-SD increase in the overall EQI were 0.21% (95% CI: –0.55%, 0.97%) to 3.16% (95% CI: 2.33%, 3.99%) for all-cause mortality, –2.73% (95% CI: –3.91%, –1.55%) to 0.54% (95% CI: –0.45%, 1.53%) for heart-disease mortality, 0.97% (95% CI: –0.46%, 2.41%) to 5.62% (95% CI: 2.82%, 8.43%) for stroke mortality, and 1.68% (95% CI: 1.48%, 1.89%) to 2.51% (95% CI: 2.35%, 2.68%) for cancer mortality.

**Table 3:** Percent difference (95% CI) in mortality rates per 1-standard-deviation increase in overall EQI from the models clustered by RUCC and by climate separately.

RUCC/Climate	All-cause	Heart disease	Stroke	Cancer
RUCC1 Metropolitan urbanized	2.28 (1.63, 2.92)	0.54 (–0.45, 1.53)	0.97 (–0.46, 2.41)	2.51 (2.35, 2.68)
RUCC2 Non-metropolitan urbanized	1.46 (0.09, 2.82)	–0.85 (–2.67, 0.98)	5.62 (2.82, 8.43)	2.05 (1.74, 2.35)
RUCC3 Less urbanized	0.21 (–0.55, 0.97)	–2.73 (–3.91, –1.55)	3.94 (2.46, 5.43)	1.68 (1.48, 1.89)
RUCC4 Thinly populated	3.16 (2.33, 3.99)	–1.44 (–2.85, –0.03)	2.25 (0.68, 3.82)	1.85 (1.53, 2.17)
Dry climate	5.30 (4.30, 6.30)	1.86 (0.04, 3.68)	12.36 (7.52, 17.21)	4.60 (2.77, 6.44)
Dry continental climate	3.70 (2.89, 4.51)	0.75 (–0.78, 2.29)	2.38 (–1.69, 6.45)	1.60 (0.22, 2.97)
Hot summer continental climate	4.24 (3.56, 4.93)	1.49 (0.22, 2.75)	–0.62 (–3.24, 2.01)	3.47 (2.43, 4.50)
Humid subtropical climate	2.29 (1.87, 2.72)	–0.50 (–1.25, 0.26)	2.65 (1.47, 3.84)	1.84 (1.27, 2.41)
Mediterranean climate	3.46 (2.32, 4.60)	1.75 (–0.04, 3.53)	4.66 (0.43, 8.89)	2.66 (0.97, 4.35)
Warm sumer continental climate	3.92 (3.15, 4.68)	0.60 (–0.70, 1.90)	–1.89 (–4.46, 0.67)	3.75 (2.70, 4.80)
Notes: CI, confidence interval; EQI, Environmental Quality Index; RUCC: Rural–Urban Continuum Code.				

For domain-specific models, we focused on all-cause mortality for brevity. The results for all-cause and cause-specific mortality can be found in Tables S4–S7. The domain-specific models clustered only by RUCC resulted in positive associations with all-cause mortality for the air and built indices across all RUCC groups, and negative or null associations for the other domains (see Table S4). The ranges of percent difference in all-cause mortality per 1-SD increase in domain-specific EQI were 2.09% (95% CI: 1.56%, 2.63%) to 3.89% (95% CI: 3.45%, 4.33%) for air, –0.57% (95% CI: –1.07%, –0.07%) to 0.16% (95% CI: –0.23%, 0.54%) for water, –1.67% (95% CI: –2.32%, –1.02%) to 0.26% (95% CI: –0.72%, 1.25%) for land, 0.69% (95% CI: 0.35%, 1.03%) to 1.38% (95% CI: 0.85%, 1.91%) for built, and –1.45% (95% CI: –2.41%, –0.49%) to 0.32% (95% CI: –0.28%, 0.93%) for sociodemographic index.

### Associations between EQI and Mortality Clustered by Climate

The hierarchical models clustered only by climate also suggested positive associations for most climate regions (Table 3). In general, the associations were higher in the dry climate for the four types of mortality studied. The ranges of percent difference in mortality rates per 1-SD increase in the overall EQI were 2.29% (95% CI: 1.87%, 2.72%) to 5.30% (95% CI: 4.30%, 6.30%) for all-cause mortality, –0.50% (95% CI: –1.25%, 0.26%) to 1.86% (95% CI: 0.04%, 3.68%) for heart disease mortality, –1.89% (95% CI: –4.46%, 0.67%) to 12.36% (95% CI: 7.52%, 17.21%) for stroke mortality, and 1.60% (95% CI: 0.22%, 2.97%) to 4.60% (95% CI: 2.77%, 6.44%) for cancer mortality.

The domain-specific models clustered only by climate showed that air had higher associations with all-cause mortality in all except the dry climate region, where the largest association was found for the land index (see Table S4). The ranges of percent difference in all-cause mortality per 1-SD increase in domain-specific EQI were –1.70% (95% CI: –3.49%, 0.08%) to 4.73% (95% CI: 3.81%, 5.66%) for air, –1.10% (95% CI: –2.30%, 0.10%) to 1.03% (95% CI: 0.28%, 1.78%) for water, –2.71% (95% CI: –3.93%, –1.50%) to 13.11% (95% CI: 9.56%, 16.66%) for land, –0.99% (95% CI: –2.10%, 0.13%) to 3.01% (95% CI: 1.45%, 4.57%) for built, and –1.66% (95% CI: –2.06%, –1.26%) to 1.51% (95% CI: 0.72%, 2.30%) for sociodemographic index.

### Associations between EQI and Mortality Clustered by RUCC–Climate Combination

**Figure 2:**
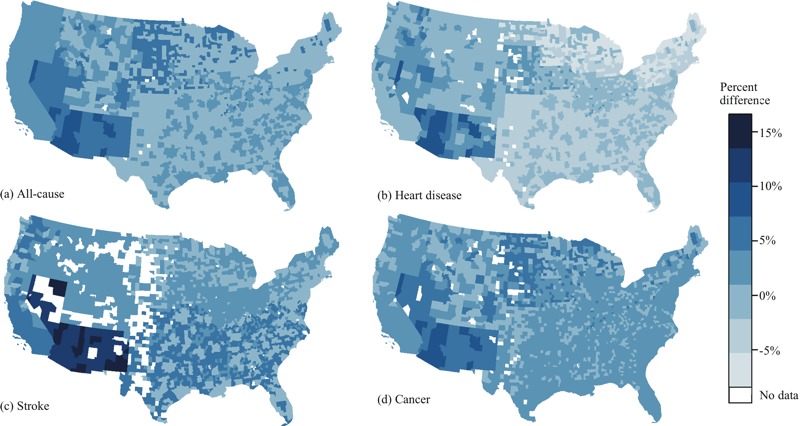
Percent difference in all-cause (*A*), heart disease (*B*), stroke (*C*), and cancer (*D*) mortality rates for 2000–2005 per 1 standard deviation increase in the overall Envirnonmental Quality Index (EQI) estimated from the models clustered by Rural–Urban Continuum Code (RUCC)–climate combination. Maps were downloaded from the U.S. Census Bureau (2010; https://www.census.gov/geo/maps-data/data/tiger.html) and were reproduced using R (version 3.0.3; R Project for Statistical Computing).

**Figure 3:**
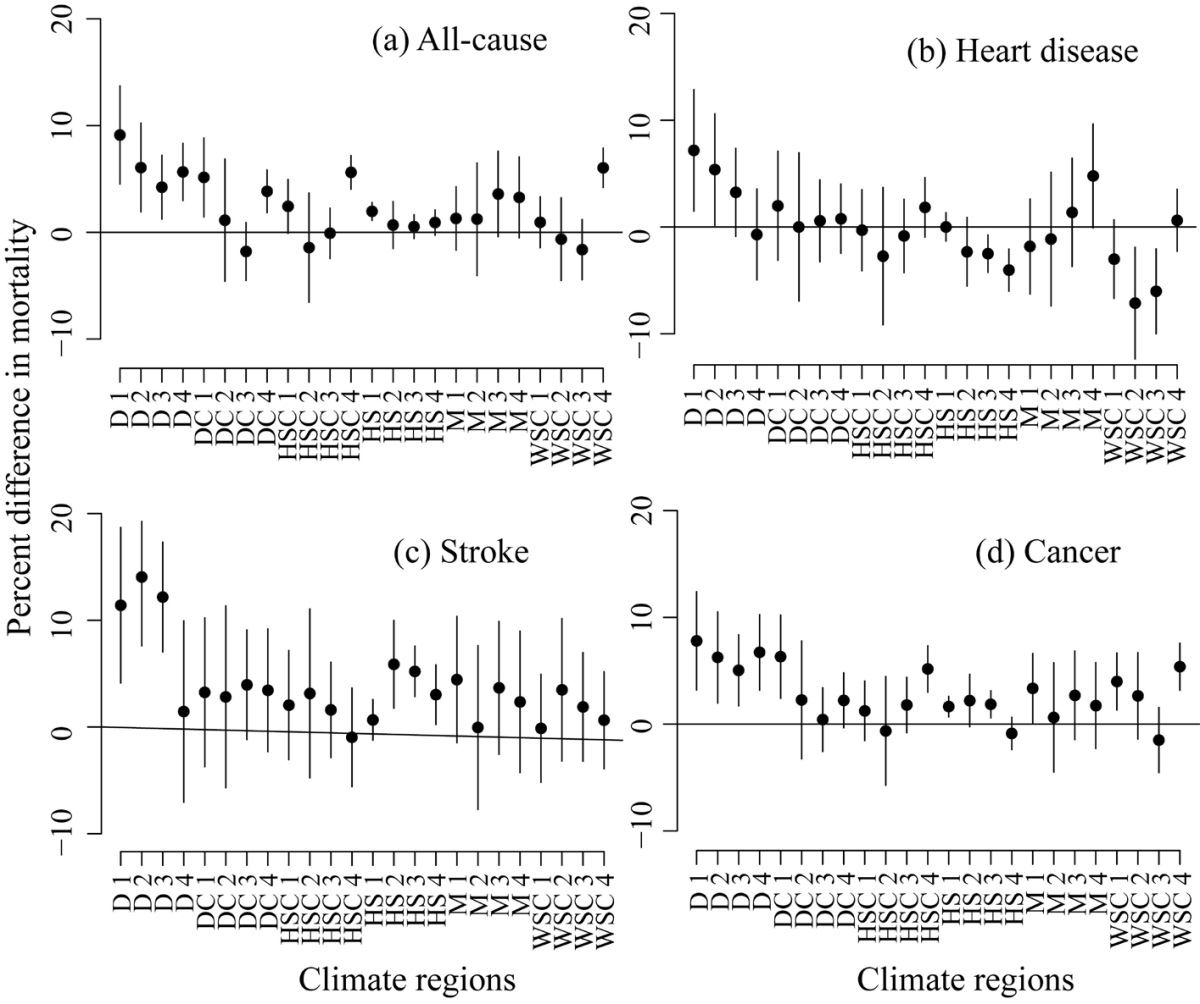
Percent difference (mean and 95% CI) in all-cause (*A*), heart disease (*B*), stroke (*C*), and cancer (*D*) mortality rates for 2000–2005 per 1 standard deviation increase in the overall Environmental Quality Index (EQI) estimated from the models clustered by Rural–Urban Climate Continuum (RUCC)–climate combination. Climate regions: dry (D), dry continental (DC), hot summer continental (HSC), humid subtropical (HS), Mediterranean (M), warm summer continental (WSC). The numbers 1–4 represent RUCC1 to RUCC4.

In general, the models for overall EQI clustered by RUCC–climate combinations reflected the spatial patterns in the models clustered by climate and RUCC separately. However, they also revealed variations related to unique combinations of RUCC–climate (Figures 2 and 3; see also Table S8). Positive or null associations between the overall EQI and mortality were observed in most of the contiguous United States.

**Figure 4:**
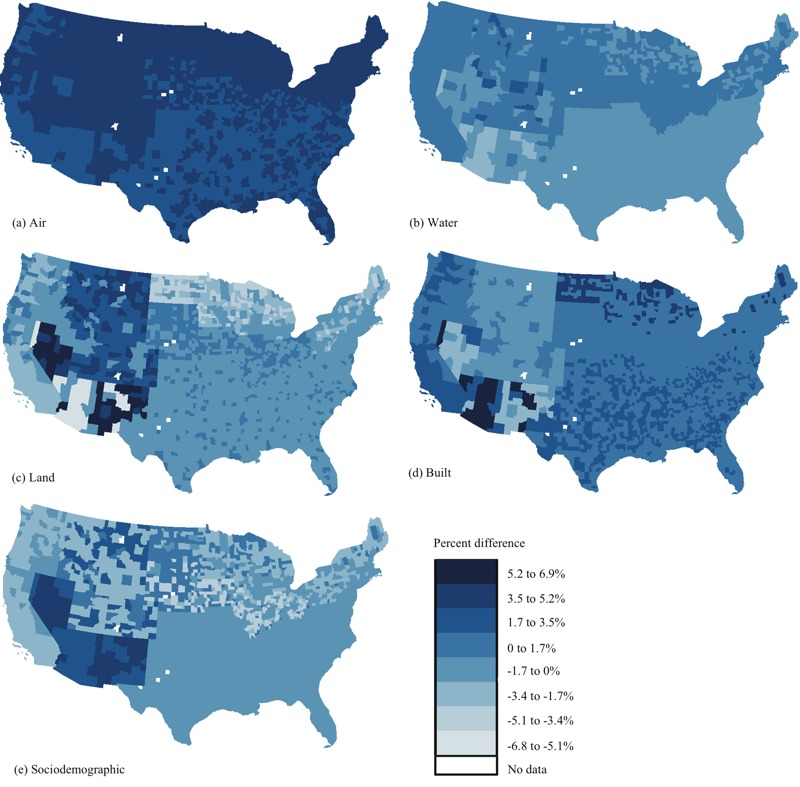
Percent difference in all-cause mortality rate for 2000–2005 per 1 standard deviation increase in the air (*A*), water (*B*), land (*C*), built (*D*), and sociodemographic (*E*) index estimated from the models clustered by Rural–Urban Climate Continuum (RUCC)–climate combination. Maps were downloaded from the U.S. Census Bureau (2010; https://www.census.gov/geo/maps-data/data/tiger.html) and were reproduced using R (version 3.0.3; R Project for Statistical Computing).

The model clustered by RUCC–climate combinations also showed that among the five domains, air had the largest association with all-cause mortality for most of the contiguous United States. (Figure 4; see also Table S9), and all the associations for air were positive. The associations between the water index and all-cause mortality were negative or null for most of the contiguous United States. Positive associations for water were mainly observed in less-populated areas (RUCC 2 to 4) in the dry continental, hot summer continental, and Mediterranean regions. Among the RUCC–climate combinations, the associations between the land index and all-cause mortality were larger in rural (RUCC3 and RUCC4) areas with dry and dry continental climates. The associations between the built index and all-cause mortality were positive for the majority of the contiguous United States. Finally, the associations between the sociodemographic index and all-cause mortality were larger in rural areas (RUCC 3 and 4) with dry climates among the RUCC–climate combinations.

## Discussion

In our analyses, we observed mostly positive associations between the overall EQI and mortality rates at the county level. Among the environmental domains, the air index had stronger associations with all-cause, heart disease, and cancer mortality compared with other domain indices, and the sociodemographic index had stronger associations with stroke mortality. The results also indicated that the associations between environmental quality and mortality vary by RUCC and climate region.

Our results were consistent with previous work, which showed significant associations between air pollutants and mortality (Zanobetti and Schwartz 2009; Kim et al. 2015). Among the five EQI domains, the air index included the most variables (*n* = 87; water *n* = 80, land *n* = 26, built *n* = 14, sociodemographic *n* = 12) and had the best data availability (Messer et al. 2014), which may contribute to a better representation of air quality. This difference in data quality across domains could also explain the heterogeneous associations between the other domains and mortality. It may also be, however, that air quality had the most influence on mortality in the United States.

In our results, large associations (> 10%) between mortality and EQI were found for some clusters. The magnitudes of these percent differences were higher than findings in previous studies involving air and land environmental quality (García-Pérez et al. 2013; Krall et al. 2013; Zanobetti and Schwartz 2009). However, our results for cumulative environmental quality cannot be directly compared with results for single environmental exposures. Furthermore, we reported the result per 1-SD increase in EQI, which may represent a considerable change in the relative cumulative environmental quality (For example, a 1-SD increase in the EQI can shift a county from being average to being in the lowest 15% of contiguous states in terms of environmental quality).

We also note that these large associations were observed mostly in the dry climate region. This region had the smallest number of counties and the largest heterogeneity in the all-cause mortality rate (Table 1; see also Table S3), which may have resulted in larger values in the estimates. A previous analysis also found that associations between fine particulate matter and mortality were stronger in the dry climate region than in other areas (Zanobetti and Schwartz 2009). Therefore, it may be that the intersection of dry climate regions and environmental quality constituted a substantial environmental burden on mortality; possible mechanisms require further investigation.

Although most of the associations between the overall EQI and mortality rates were positive, negative associations were still observed in the analysis, particularly for heart disease mortality and for the water, land, and sociodemographic indices. These negative associations represented counterintuitive results suggesting that worse environmental quality was linked with lower mortality rates. One possible explanation for the observed negative associations was the spatial scale used in the models. Analysis based on county-level exposure data may be too diffuse for areas with high heterogeneity in population distribution or environmental quality (Messer et al. 2014; Rappazzo et al. 2015). Additionally, the county level is not the optimal unit of geographic aggregation for all of the domains contained within the EQI. The amount of spatial variability within a county may differ among environmental domains. If the particular variables within a domain are homogeneous at the county level, they will be better represented than variables that are more heterogeneous, which could be a form of exposure misclassification. This misclassification may lead to potentially lower effect estimates in the more urban areas where more heterogeneity is expected. EQIs at finer spatial scales (e.g., at the census-tract level) and studies that explore environmental effects at multiple spatial scales may shed light on these counterintuitive results.

Because this research is novel, it is exploratory in nature and is not driven by *a priori* hypotheses. In our analysis, we explored the spatial patterns in the associations between mortality and EQI by RUCC, by climate, and by the combination of RUCC and climate. The hierarchical models shrank the estimated associations for each cluster toward the average associations for the contiguous United States (partial pooling). This shrinkage made comparisons among the clusters more conservative than stratified analysis (Gelman et al. 2012). In lieu of null hypothesis testing, we examined patterns of the estimated associations between EQI and mortality and focused on their magnitude and precision (95% CI) rather than on their statistical significance, thereby not raising multiple testing concerns.

A limitation of the analysis was its ecological design and cross-sectional nature. In this study, we associated 2000–2005 EQI with mortality rates in the same period. Thus, the results were likely to represent the health impact of environmental quality in a relatively short time period. We believe that this focus on cross-sectional associations was appropriate, considering the short-term associations between environmental quality and mortality reported in many of the previous studies, in particular studies of air quality (Ostro et al. 2011; Tao et al. 2012; Zanobetti and Schwartz 2009). Additionally, if the county-level environmental quality remained relatively stable, such that the 2000–2005 EQI also reflected the county-level environmental quality of previous years, the associations in this study can be generalized to health impacts of environmental quality outside the 6-year time period. An analysis using current EQI and delayed mortality rates may help reveal long-term effects.

In this study, we used the EQI and its domain indices as continuous variables in models, assuming a linear relationship between the EQI and the natural log of the mortality rates. This modeling decision may be considered another potential limitation of our analysis because environmental quality can exhibit complex nonlinear impacts on mortality. However, our exploratory graphical analysis did not reveal apparent deviations from linearity (results not shown). Furthermore, the assumption of linearity leads to straightforward interpretation of the results. The linear model that we used can also represent the average association of EQI with ln(mortality) across the environmental quality gradient and thus can reflect the general trend in the relationship. So, although it is limiting, we viewed this approach to be the most appropriate one to use for this exploratory analysis.

A key strength of this study was the use of the EQI to represent cumulative environmental quality. It combined multiple domains at the county level and provided coverage for the entire United States, both of which represent improvements over other environmental indices (Messer et al. 2014). Compared with previous studies using single exposures, the use of EQI was more likely to capture the health effects resulting from the overall burden of environmental exposures. The five domains of EQI also provided a way to compare the health impacts of different aspects of environmental quality. To our knowledge, this is the first study in the United States to assess cumulative environmental effects on mortality.

Another major strength of this work was its national scale and its inclusion of spatial heterogeneity via urbanicity and climate. The three types of regional clustering (RUCC, climate, and RUCC–climate combined) allowed us to explore the spatial patterns in the associations between EQI and mortality across the contiguous United States. Compared with studies restricted to a smaller spatial scale, and compared with studies at the national level but without spatial heterogeneity, this study has the potential to show differences in environmental impacts on mortality, which may offer information for prioritizing efforts in addressing environmental problems.

## Conclusions

This study was the first attempt to assess the cumulative environmental impact on mortality and its spatial patterns in the United States. We found positive associations for the majority of the contiguous United States, suggesting adverse effects of poor cumulative environmental quality. We found that among the five EQI domain indices, air had the largest associations with all-cause, heart disease, and cancer mortality, whereas the sociodemographic index had the largest association with stroke mortality. The associations varied for urbanicity and climate regions, suggesting different environmental impacts on mortality across the contiguous United States. In particular, large associations were found in the dry climate region. This work demonstrated the use of EQI as a useful tool to assess the cumulative environmental burden at the county level in addition to demonstrating the use of the five domain indices of EQI to assess the co-occurring environmental impacts.

## Supplemental Material

(307 KB) PDF

## References

[r1] Bates D, Mächler M, Bolker B, Walker S (2015). Fitting linear mixed-effects models using lme4.. J Stat Softw.

[r2] CDC (Centers for Disease Control and Prevention) (2015). CDC Wonder. WONDER Search.. http://wonder.cdc.gov/.

[r3] Cossman JS, James WL, Cosby AG, Cossman RE (2010). Underlying causes of the emerging nonmetropolitan mortality penalty.. Am J Public Health.

[r4] Curtin LR, Klein RJ (1995). Direct Standardization (Age-Adjusted Death Rates) Healthy People 2000, Statistical Notes (No. 6–Revised). DHHS Publication No. (PHS) 95–1237.. https://www.cdc.gov/Nchs/data/statnt/statnt06rv.pdf.

[r5] Dwyer-Lindgren L, Flaxman AD, Ng M, Hansen GM, Murray CJL, Mokdad AH (2015). Drinking patterns in US counties from 2002 to 2012.. Am J Public Health.

[r6] Dwyer-Lindgren L Mokdad AH Srebotnjak T Flaxman AD Hansen GM Murray CJL 2014 Cigarette smoking prevalence in US counties: 1996–2012. Popul Health Metr 12 5, doi:10.1186/1478-7954-12-5 24661401 PMC3987818

[r7] Ezzati M Friedman AB Kulkarni SC Murray CJL 2008 The reversal of fortunes: trends in county mortality and cross-county mortality disparities in the United States. PLoS Med 5 e66, doi:10.1371/journal.pmed.0050066 18433290 PMC2323303

[r8] García-Pérez J, Fernández-Navarro P, Castelló A, López-Cima MF, Ramis R, Boldo E (2013). Cancer mortality in towns in the vicinity of incinerators and installations for the recovery or disposal of hazardous waste.. Environ Int.

[r9] Gelman A Hill J Yajima M 2012 Why We (Usually) Don’t Have to Worry About Multiple Comparisons. J Res Educ Eff 5 2 189 211, doi: 10.1080/19345747.2011.618213

[r10] Harmon MP, Coe K (1993). Cancer mortality in U.S. counties with hazardous waste sites.. Popul Environ.

[r11] Hendryx M Conley J Fedorko E Luo J Armistead M 2012 Permitted water pollution discharges and population cancer and non-cancer mortality: toxicity weights and upstream discharge effects in US rural-urban areas. Int J Health Geogr 11 9, doi:10.1186/1476-072X-11-9 22471926 PMC3342919

[r12] Heron M (2013). Deaths: leading causes for 2010.. Natl Vital Stat Rep.

[r13] Kim SE, Lim YH, Kim H (2015). Temperature modifies the association between particulate air pollution and mortality: a multi-city study in South Korea.. Sci Total Environ.

[r14] Kottek M, Grieser J, Beck C, Rudolf B, Rubel F (2006). World map of the Köppen-Geiger climate classification updated.. Meteorol Z.

[r15] Krall JR Anderson GB Dominici F Bell ML Peng RD 2013 Short-term exposure to particulate matter constituents and mortality in a national study of U.S. urban communities. Environ Health Perspect 121 1148 1153, doi:10.1289/ehp.1206185 23912641 PMC3801200

[r16] Langlois PH, Jandle L, Scheuerle A, Horel SA, Carozza SE (2010). Occurrence of conotruncal heart birth defects in Texas: a comparison of urban/rural classifications.. J Rural Health.

[r17] Lobdell DT, Jagai JS, Rappazzo K, Messer LC (2011). Data sources for an environmental quality index: availability, quality, and utility.. Am J Public Health.

[r18] Lu F, Zhou L, Xu Y, Zheng TZ, Guo YM, Wellenius GA (2015). Short-term effects of air pollution on daily mortality and years of life lost in Nanjing, China.. Sci Total Environ.

[r19] Luben TJ, Messer LC, Mendola P, Carozza SE, Horel SA, Langlois PH (2009). Urban–rural residence and the occurrence of neural tube defects in Texas, 1999–2003.. Health Place.

[r20] Messer LC Jagai JS Rappazzo KM Lobdell DT 2014 Construction of an environmental quality index for public health research. Environ Health 13 39, doi:10.1186/1476-069X-13-39 24886426 PMC4046025

[r21] Messer LC, Luben TJ, Mendola P, Carozza SE, Horel SA, Langlois PH (2010). Urban-rural residence and the occurrence of cleft lip and cleft palate in Texas, 1999–2003.. Ann Epidemiol.

[r22] Nordio F, Zanobetti A, Colicino E, Kloog I, Schwartz J (2015). Changing patterns of the temperature–mortality association by time and location in the US, and implications for climate change.. Environ Int.

[r23] O’Neill MS, Zanobetti A, Schwartz J (2005). Disparities by race in heat-related mortality in four US cities: the role of air conditioning prevalence.. J Urban Health.

[r24] Ostro B Tobias A Cluerol X Alastuey A Amato F Pey J 2011 The effects of particulate matter sources on daily mortality: a case-crossover study of Barcelona, Spain. Environ Health Perspect 119 1781 1787, doi:10.1289/ehp.1103618 21846610 PMC3261985

[r25] Pearce JR, Richardson EA, Mitchell RJ, Shortt NK (2010). Environmental justice and health: the implications of the socio-spatial distribution of multiple environmental deprivation for health inequalities in the United Kingdom.. Trans Inst Br Geogr.

[r26] Pearce JR, Richardson EA, Mitchell RJ, Shortt NK (2011). Environmental justice and health: a study of multiple environmental deprivation and geographical inequalities in health in New Zealand.. Soc Sci Med.

[r27] Rappazzo KM Messer LC Jagai JS Gray CL Grabich SC Lobdell DT 2015 The associations between environmental quality and preterm birth in the United States, 2000–2005: a cross-sectional analysis. Environ Health 14 1 50, doi:10.1186/s12940-015-0038-3 26051702 PMC4464856

[r28] Ren H, Wan X, Yang F, Shi X, Xu J, Zhuang D (2015). Association between changing mortality of digestive tract cancers and water pollution: a case study in the Huai River Basin, China.. Int J Environ Res Public Health.

[r29] Schwartz J, Austin E, Bind MA, Zanobetti A, Koutrakis P (2015). Estimating causal associations of fine particles with daily deaths in Boston.. Am J Epidemiol.

[r30] Tao Y Huang W Huang X Zhong L Lu SE Li Y 2012 Estimated acute effects of ambient ozone and nitrogen dioxide on mortality in the Pearl River Delta of southern China. Environ Health Perspect 120 393 398, doi:10.1289/ehp.1103715 22157208 PMC3295344

[r31] Turner MC, Krewski D, Chen Y, Pope CA, Gapstur SM, Thun MJ (2012a). Radon and COPD mortality in the American Cancer Society cohort.. Eur Respir J.

[r32] Turner MC, Krewski D, Chen Y, Pope CA, Gapstur SM, Thun MJ (2012b). Radon and nonrespiratory mortality in the American Cancer Society cohort.. Am J Epidemiol.

[r33] U.S. Census Bureau (2010). TIGER Products.. https://www.census.gov/geo/maps-data/data/tiger.html.

[r34] U.S. Census Bureau (2012). Population Estimates.. http://www.census.gov/popest/data/intercensal/.

[r35] U.S. EPA (U. S. Environmental Protection Agency) (2014). Creating an Overall Environmental Quality Index – Technical Report. EPA/600/R-14/304.. https://edg.epa.gov/data/Public/ORD/NHEERL/EQI.

[r36] U.S. EPA (2015). EPA Environmental Dataset Gateway Download Locations.. https://edg.epa.gov/data/Public/ORD/NHEERL/EQI.

[r37] USDA (U.S. Department of Agriculture) (2015). Rural-Urban Continuum Codes.. http://www.ers.usda.gov/data-products/rural-urban-continuum-codes/documentation.aspx.

[r38] Zanobetti A Schwartz J 2009 The effect of fine and coarse particulate air pollution on mortality: a national analysis. Environ Health Perspect 117 898 903, doi:10.1289/ehp.0800108 19590680 PMC2702403

