# Supplementary material for: Associations between Environmental Quality and Mortality in the Contiguous United States, 2000–2005
Source: Environ Health Perspect. 2016 Oct 7;125(3):355–62. doi: 10.1289/EHP119 (PMC5332172; doi:10.1289/EHP119)
Supplement: (307 KB) PDF [file EHP119.s001.acco.pdf]

Environ Health Perspect
DOI: 10.1289/EHP119

Note to readers with disabilities: *EHP* strives to ensure that all journal content is accessible to all readers. However, some figures and Supplemental Material published in *EHP* articles may not conform to 508 standards due to the complexity of the information being presented. If you need assistance accessing journal content, please contact ehponline@niehs.nih.gov. Our staff will work with you to assess and meet your accessibility needs within 3 working days.

Supplemental Material

The Associations between Environmental Quality and Mortality in the Contiguous United States, 2000-2005

Yun Jian, Lynne C. Messer, Jyotsna S. Jagai, Kristen M. Rappazzo, Christine L. Gray, Shannon C. Grabich, and Danelle T. Lobdell

Table of Contents

Table S1. Mean, Standard deviation (SD), and range of EQI indices 2000-2005 for the contiguous U.S.
Table S2. States in the condensed climate regions in the contiguous U.S.
Table S3. Number of counties in each RUCC-climate region combination in the contiguous U.S. 2000-2005
Table S4. Percent difference (and 95%CI) in all-cause mortality rate 2000-2005 per 1 standard deviations increase in EQI domain indices estimated from the models clustered by RUCC and climate regions separately
Table S5. Percent difference (and 95%CI) in heart disease mortality rates 2000-2005 per 1 standard deviations increase in EQI domain indices estimated from the models clustered by RUCC and climate regions separately.
Table S6. Percent difference (95%CI) in stroke mortality rates for the year 2000-2005 per 1 standard deviations increase in EQI domain indices estimated from the models clustered by RUCC and climate regions separately.

Table S7. Percent difference (95%CI) in cancer mortality rates 2000-20005 per 1 standard deviations increase in EQI domain indices estimated from the models clustered by RUCC and climate regions separately.

Table S8. Percent difference (and 95%CI) in all-cause and cause-specific mortality rates 2000-2005 per 1 standard deviations increase in the overall EQI estimated from the model clustered by RUCC-climate combination.

Table S9. Percent difference (and 95%CI) in all-cause and cause-specific mortality rates per 1 standard deviations increase in EQI domain indices estimated from the model clustered by RUCC-climate combination.

Table S1. Mean, Standard deviation (SD), and range of EQI 2000-2005 indices for the contiguous U.S.

EQI	Mean	SD	Min	Max
Overall EQI	0.03	0.96	-4.22	2.85
Air	0.02	0.98	-3.24	2.79
Water	0.00	1.00	-1.64	1.48
Land	0.05	0.86	-5.14	2.09
Built	0.01	0.98	-6.06	3.88
Sociodemographic	0.01	0.99	-4.81	3.98

Table S2. States in the condensed climate regions in the contiguous U.S.

Climate regions	States
Dry	New Mexico, Arizona, Nevada
Dry continental	Montana, Idaho, Wyoming, Utah, Colorado
Hot summer continental	South Dakota, Nebraska, Iowa, Illinois, Indiana, Ohio
Humid subtropical	Florida, Louisiana, Texas, Georgia, Alabama, Mississippi, Arkansas, Oklahoma, Kansas, Missouri, Tennessee, South Carolina, North Carolina, Virginia, West Virginia, Kentucky
Mediterranean	California, Oregon, Washington
Warm summer continental	North Dakota, Minnesota, Wisconsin, Michigan, Pennsylvania, New York, Connecticut, Rhode Island, Massachusetts, Vermont, New Hampshire, Maine

Table S3. Number of counties in each RUCC-climate region combination in the contiguous U.S. 2000-2005

Number of counties	Dry	Dry continental	Hot summer continental	Humid subtropical	Mediterranean	Warm sumer continental
RUCC 1	17	44	158	623	65	178
RUCC 2	13	15	61	144	21	65
RUCC 3	26	84	191	571	32	145
RUCC 4	9	72	130	327	15	103

Table S4. Percent difference (and 95%CI) in all-cause mortality rate 2000-2005 per 1 standard deviations increase in EQI domain indices estimated from the models clustered by RUCC and climate regions separately

**RUCC**	**Air**	**Water**	**Land**	**Built**	**Sociodemographic**
RUCC1	3.89 (3.45,4.33)	-0.44 (-0.80,-0.08)	-1.03 (-1.62,-0.44)	0.69 (0.35,1.03)	0.03 (-0.43,0.49)
RUCC2	3.37 (2.47,4.28)	-0.57 (-1.07,-0.07)	0.26 (-0.72,1.25)	1.38 (0.85,1.91)	-1.45 (-2.41,-0.49)
RUCC3	2.09 (1.56,2.63)	-0.41 (-0.76,-0.05)	-1.67 (-2.32,-1.02)	1.18 (0.84,1.51)	-1.08 (-1.63,-0.52)
RUCC4	3.26 (2.74,3.78)	0.16 (-0.23,0.54)	-1.58 (-2.37,-0.79)	1.11 (0.75,1.46)	0.32 (-0.28,0.93)
**Climate**	**Air**	**Water**	**Land**	**Built**	**Sociodemographic**
Dry	-1.70 (-3.49,0.08)	-1.10 (-2.30,0.10)	13.11 (9.56,16.66)	3.01 (1.45,4.57)	1.51 (0.72,2.30)
Dry continental	4.60 (3.53,5.67)	0.13 (-0.90,1.16)	2.74 (-0.27,5.75)	-0.99 (-2.10,0.13)	-0.92 (-1.37,-0.47)
Hot summer continental	4.26 (3.42,5.10)	1.03 (0.28,1.78)	-1.50 (-3.33,0.34)	0.74 (0.15,1.33)	-1.36 (-1.71,-1.01)
Humid subtropical	2.56 (1.98,3.14)	-0.58 (-0.97,-0.19)	-0.94 (-1.37,-0.51)	1.41 (1.03,1.78)	-0.31 (-0.57,-0.04)
Mediterranean	2.79 (0.98,4.61)	0.57 (-0.34,1.48)	0.73 (-4.79,6.25)	2.19 (0.93,3.46)	-0.87 (-1.72,-0.03)
Warm summer continental	4.73 (3.81,5.66)	0.11 (-0.54,0.76)	-2.71 (-3.93,-1.50)	2.02 (1.12,2.92)	-1.66 (-2.06,-1.26)

RUCC1 : Metropolitan urbanized, RUCC2: Non-metropolitan urbanized, RUCC3: Less urbanized, RUCC 4: Thinly populate

DOI: 10.1289/EHP119

Table S5. Percent difference (and 95%CI) in heart disease mortality rates 2000-2005 per 1 standard deviations increase in EQI domain indices estimated from the models clustered by RUCC and climate regions separately.

RUCC	Air	Water	Land	Built	Sociodemographic
RUCC1	4.91 (4.13,5.69)	-1.39 (-1.78,-1.00)	-1.16 (-2.06,-0.27)	-1.03 (-1.20,-0.86)	-1.86 (-2.54,-1.19)
RUCC2	7.55 (6.06,9.03)	-2.66 (-3.34,-1.98)	0.80 (-0.86,2.47)	-0.55 (-0.82,-0.27)	-3.90 (-5.03,-2.77)
RUCC3	5.33 (4.41,6.25)	-2.28 (-2.76,-1.80)	-2.75 (-3.57,-1.94)	-0.55 (-0.75,-0.36)	-3.67 (-4.47,-2.86)
RUCC4	3.47 (2.57,4.36)	-0.91 (-1.46,-0.37)	-2.84 (-3.63,-2.06)	-1.17 (-1.41,-0.93)	-1.23 (-2.19,-0.26)
**Climate**	**Air**	**Water**	**Land**	**Built**	**Sociodemographic**
Dry	-6.45 (-9.72,-3.18)	1.06 (-1.16,3.28)	10.79 (8.35,13.23)	0.35 (-1.26,1.95)	4.35 (2.31,6.39)
Dry continental	3.93 (1.95,5.92)	0.50 (-1.32,2.31)	2.90 (0.49,5.31)	-2.40 (-3.44,-1.37)	-2.42 (-4.05,-0.80)
Hot summer continental	4.13 (2.67,5.59)	-0.42 (-1.69,0.85)	-0.36 (-1.80,1.09)	-1.08 (-2.03,-0.14)	-2.93 (-4.04,-1.83)
Humid subtropical	2.67 (1.80,3.54)	-1.43 (-2.08,-0.77)	-0.97 (-1.39,-0.54)	0.52 (-0.11,1.14)	-3.22 (-3.77,-2.68)
Mediterranean	3.51 (0.52,6.51)	1.66 (0.17,3.14)	2.67 (0.03,5.31)	-2.11 (-3.52,-0.70)	-2.72 (-4.90,-0.54)
Warm summer continental	8.24 (6.77,9.70)	-2.52 (-3.58,-1.46)	-3.35 (-4.55,-2.15)	-1.85 (-2.80,-0.90)	-5.71 (-6.79,-4.63)

Table S6. Percent difference (95%CI) in cancer mortality rates 2000-20005 per 1 standard deviations increase in EQI domain indices estimated from the models clustered by RUCC and climate regions separately.

RUCC	Air	Water	Land	Built	Sociodemographic
RUCC1	3.30 (3.10,3.50)	-0.74 (-1.22,-0.26)	-0.78 (-1.21,-0.35)	0.04 (-0.29,0.37)	0.90 (0.45,1.35)
RUCC2	3.24 (2.96,3.52)	-0.71 (-1.38,-0.03)	0.16 (-0.60,0.91)	1.28 (0.44,2.13)	-0.43 (-1.38,0.52)
RUCC3	3.30 (3.15,3.46)	-0.77 (-1.13,-0.40)	-0.73 (-1.22,-0.24)	0.37 (-0.15,0.88)	0.51 (-0.11,1.12)
RUCC4	2.85 (2.66,3.05)	0.32 (-0.14,0.78)	-0.57 (-1.08,-0.07)	0.30 (-0.19,0.79)	0.09 (-0.55,0.73)
**Climate**	**Air**	**Water**	**Land**	**Built**	**Sociodemographic**
Dry	0.56 (-0.03,1.15)	-1.73 (-3.02,-0.45)	11.07 (7.94,14.20)	0.00 (-2.01,2.02)	2.85 (1.88,3.81)
Dry continental	2.79 (2.18,3.41)	0.49 (-0.54,1.51)	3.59 (0.91,6.26)	-1.97 (-3.22,-0.71)	-0.26 (-1.17,0.64)
Hot summer continental	2.81 (2.40,3.21)	0.27 (-0.37,0.91)	-0.57 (-2.33,1.19)	0.36 (-0.69,1.41)	0.02 (-0.60,0.65)
Humid subtropical	2.76 (2.61,2.91)	-0.67 (-1.04,-0.30)	-0.12 (-0.57,0.33)	-0.12 (-0.59,0.34)	0.25 (-0.02,0.51)
Mediterranean	2.61 (1.79,3.43)	-0.59 (-1.67,0.49)	-2.48 (-6.90,1.94)	1.81 (-0.05,3.67)	0.60 (-0.53,1.72)
Warm summer continental	2.56 (2.16,2.96)	0.48 (-0.10,1.07)	-1.61 (-2.71,-0.50)	1.88 (0.72,3.04)	0.40 (-0.22,1.02)

Environ Health Perspect
DOI: 10.1289/EHP119

Table S7. Percent difference (95%CI) in stroke mortality rates 2000-2005 per 1 standard deviations increase in EQI domain indices estimated from the models clustered by RUCC and climate regions separately.

RUCC	Air	Water	Land	Built	Sociodemographic
RUCC1	0.36 (-0.81,1.54)	0.64 (0.49,0.79)	0.45 (-0.03,0.93)	0.43 (-0.10,0.96)	1.83 (0.52,3.14)
RUCC2	-0.03 (-2.27,2.21)	0.70 (0.41,0.99)	-0.68 (-1.79,0.43)	0.91 (-0.26,2.07)	4.40 (1.80,7.00)
RUCC3	2.27 (0.56,3.98)	0.41 (0.20,0.63)	-1.87 (-2.60,-1.14)	2.54 (1.63,3.45)	5.37 (3.76,6.98)
RUCC4	-4.49 (-6.22,-2.77)	1.28 (1.06,1.50)	-1.39 (-2.36,-0.42)	-0.55 (-1.46,0.36)	8.73 (6.47,10.99)
**Climate**	**Air**	**Water**	**Land**	**Built**	**Sociodemographic**
Dry	-0.12 (-4.55,4.32)	-18.58 (-23.06,-14.09)	6.82 (1.00,12.63)	1.32 (-1.45,4.08)	11.25 (8.79,13.70)
Dry continental	1.74 (-1.25,4.74)	-3.31 (-7.52,0.90)	4.48 (0.79,8.18)	-0.55 (-2.72,1.61)	3.00 (0.28,5.72)
Hot summer continental	0.51 (-1.17,2.19)	0.06 (-2.08,2.20)	1.95 (-0.06,3.96)	-1.53 (-3.40,0.34)	1.48 (-0.23,3.18)
Humid subtropical	-1.71 (-2.59,-0.83)	1.73 (0.77,2.70)	-0.87 (-1.92,0.18)	1.72 (0.85,2.60)	3.55 (2.80,4.31)
Mediterranean	2.97 (0.58,5.36)	-1.89 (-4.86,1.09)	5.73 (2.75,8.72)	1.51 (-0.93,3.96)	3.14 (0.95,5.33)
Warm summer continental	-4.80 (-6.72,-2.88)	0.19 (-1.67,2.06)	-4.29 (-6.39,-2.19)	-0.12 (-1.67,1.42)	4.30 (2.56,6.05)

DOI: 10.1289/EHP119

Table S8. Percent difference (and 95%CI) in all-cause and cause-specific mortality rates 2000-2005 per 1 standard deviations increase in the overall EQI estimated from the model clustered by RUCC-climate combination.

Climates	RUCC	All-cause	Heart disease	Cancer	Stroke
Dry	RUCC1	9.12 (4.52,13.72)	7.18 (1.48,12.87)	7.80 (3.19,12.40)	11.41 (4.12,18.70)
	RUCC2	6.07 (1.91,10.23)	5.38 (0.15,10.60)	6.25 (1.97,10.52)	14.05 (7.60,20.50)
	RUCC3	4.23 (1.23,7.22)	3.24 (-0.88,7.37)	5.04 (1.71,8.37)	12.17 (7.03,17.31)
	RUCC4	5.66 (2.98,8.33)	-0.70 (-4.95,3.56)	6.72 (3.18,10.26)	1.45 (-7.05,9.95)
Dry continental	RUCC1	5.14 (1.44,8.84)	1.99 (-3.11,7.09)	6.32 (2.42,10.22)	3.25 (-3.72,10.22)
	RUCC2	1.14 (-4.59,6.86)	0.00 (-6.94,6.95)	2.27 (-3.25,7.78)	2.83 (-5.70,11.35)
	RUCC3	-1.81 (-4.52,0.91)	0.57 (-3.26,4.40)	0.42 (-2.56,3.41)	3.95 (-1.19,9.09)
	RUCC4	3.85 (1.84,5.85)	0.78 (-2.46,4.01)	2.22 (-0.35,4.80)	3.43 (-2.32,9.19)
Hot summer continental	RUCC1	2.43 (-0.10,4.95)	-0.30 (-4.10,3.50)	1.24 (-1.55,4.03)	2.05 (-3.06,7.16)
	RUCC2	-1.44 (-6.56,3.69)	-2.73 (-9.16,3.70)	-0.64 (-5.74,4.46)	3.15 (-4.77,11.06)
	RUCC3	-0.09 (-2.45,2.26)	-0.84 (-4.28,2.59)	1.78 (-0.81,4.38)	1.61 (-2.87,6.08)
	RUCC4	5.62 (4.04,7.19)	1.84 (-0.95,4.63)	5.16 (2.98,7.34)	-0.97 (-5.59,3.66)
Humid subtropical	RUCC1	1.96 (1.11,2.80)	0.01 (-1.33,1.34)	1.64 (0.68,2.59)	0.67 (-1.23,2.57)
	RUCC2	0.68 (-1.52,2.88)	-2.32 (-5.54,0.90)	2.21 (-0.25,4.66)	5.87 (1.76,9.98)
	RUCC3	0.53 (-0.59,1.64)	-2.50 (-4.24,-0.75)	1.86 (0.58,3.14)	5.21 (2.85,7.58)
	RUCC4	0.91 (-0.29,2.11)	-4.04 (-6.02,-2.07)	-0.88 (-2.39,0.64)	3.03 (0.24,5.81)
Mediterranean	RUCC1	1.30 (-1.67,4.26)	-1.83 (-6.28,2.62)	3.35 (0.08,6.61)	4.44 (-1.48,10.37)
	RUCC2	1.23 (-4.05,6.50)	-1.14 (-7.40,5.13)	0.63 (-4.49,5.75)	-0.05 (-7.72,7.62)
	RUCC3	3.59 (-0.41,7.59)	1.37 (-3.70,6.44)	2.70 (-1.46,6.85)	3.66 (-2.57,9.90)
	RUCC4	3.27 (-0.53,7.06)	4.78 (-0.09,9.65)	1.74 (-2.28,5.76)	2.36 (-4.28,8.99)
Warm summer continental	RUCC1	0.94 (-1.45,3.33)	-3.01 (-6.69,0.67)	3.99 (1.32,6.67)	-0.13 (-5.18,4.92)
	RUCC2	-0.65 (-4.53,3.24)	-7.14 (-12.38,-1.89)	2.65 (-1.40,6.70)	3.48 (-3.20,10.16)
	RUCC3	-1.62 (-4.44,1.20)	-6.03 (-10.01,-2.06)	-1.49 (-4.55,1.57)	1.88 (-3.21,6.98)
	RUCC4	6.05 (4.20,7.90)	0.63 (-2.27,3.53)	5.37 (3.17,7.57)	0.63 (-3.92,5.18)

Environ Health Perspect
DOI: 10.1289/EHP119

Table S9. Percent difference (and 95%CI) in all-cause and cause-specific mortality rates 2000-2005 per 1 standard deviations increase in EQI domain indices estimated from the model clustered by RUCC-climate combination.

Air		All-cause	Heart	Cancer	Stroke
Dry	RUCC1	1.74 (-0.37,3.85)	-5.64 (-12.35,1.07)	0.71 (-1.39,2.80)	-3.31 (-9.85,3.22)
	RUCC2	3.15 (0.82,5.48)	-2.82 (-9.38,3.73)	1.78 (-0.53,4.10)	-2.76 (-10.14,4.62)
	RUCC3	3.39 (1.34,5.44)	-5.97 (-11.26,-0.68)	0.00 (-2.26,2.26)	-0.98 (-7.03,5.07)
	RUCC4	4.18 (2.11,6.25)	-8.24 (-16.69,0.21)	2.09 (-0.48,4.66)	-1.52 (-9.10,6.05)
Dry continental	RUCC1	3.38 (1.42,5.34)	1.62 (-3.43,6.67)	1.58 (-0.41,3.57)	1.80 (-3.25,6.86)
	RUCC2	4.85 (2.59,7.10)	6.17 (-0.51,12.85)	2.80 (0.45,5.14)	1.70 (-4.98,8.38)
	RUCC3	3.84 (2.34,5.34)	6.51 (3.10,9.92)	2.88 (0.96,4.80)	4.14 (-0.92,9.20)
	RUCC4	4.85 (3.57,6.12)	4.03 (0.38,7.67)	1.75 (-0.27,3.76)	-3.38 (-8.86,2.09)
Hot summer continental	RUCC1	3.78 (2.36,5.20)	4.71 (1.40,8.03)	2.41 (1.15,3.67)	1.76 (-1.53,5.06)
	RUCC2	3.73 (1.83,5.62)	6.23 (0.95,11.51)	2.55 (0.98,4.13)	0.99 (-3.47,5.45)
	RUCC3	2.73 (1.30,4.15)	5.67 (2.89,8.46)	2.22 (0.82,3.61)	1.90 (-2.03,5.83)
	RUCC4	4.69 (3.65,5.74)	1.88 (-1.11,4.87)	3.46 (1.77,5.16)	-4.17 (-8.11,-0.24)
Humid subtropical	RUCC1	3.98 (3.17,4.79)	3.03 (1.22,4.85)	3.90 (3.27,4.52)	-0.47 (-2.02,1.09)
	RUCC2	2.87 (1.25,4.48)	4.39 (0.92,7.86)	3.09 (1.67,4.51)	0.06 (-3.14,3.25)
	RUCC3	1.72 (0.68,2.77)	2.57 (0.84,4.31)	3.82 (2.87,4.77)	-0.27 (-2.66,2.12)
	RUCC4	2.23 (1.30,3.16)	2.22 (0.54,3.89)	1.03 (-0.16,2.23)	-6.76 (-10.04,-3.48)
Mediterranean	RUCC1	3.11 (1.42,4.81)	2.73 (-1.48,6.94)	2.74 (1.16,4.32)	3.81 (0.27,7.36)
	RUCC2	3.67 (1.53,5.81)	-2.15 (-9.69,5.40)	3.34 (1.20,5.47)	6.08 (0.45,11.71)
	RUCC3	3.67 (1.76,5.58)	-1.43 (-7.50,4.65)	2.37 (0.09,4.66)	1.10 (-5.03,7.23)
	RUCC4	4.06 (2.03,6.08)	8.52 (-1.12,18.16)	2.76 (0.19,5.33)	-0.63 (-8.39,7.13)
Warm summer continental	RUCC1	3.37 (1.96,4.79)	7.96 (4.86,11.05)	2.30 (1.33,3.26)	-4.63 (-7.10,-2.17)
	RUCC2	3.98 (1.94,6.02)	13.44 (8.19,18.69)	1.81 (0.37,3.24)	-3.69 (-8.04,0.66)
	RUCC3	3.99 (2.23,5.74)	13.12 (9.27,16.98)	2.36 (0.84,3.87)	-0.03 (-4.59,4.54)
	RUCC4	4.60 (3.45,5.76)	5.45 (2.12,8.78)	4.08 (2.42,5.74)	-1.12 (-6.00,3.76)
**Water**		**All-cause**	**Heart**	**Cancer**	**Stroke**
Dry	RUCC1	-3.09 (-4.76,-1.41)	1.56 (-0.29,3.42)	-1.39 (-2.46,-0.32)	-6.38 (-10.73,-2.03)
	RUCC2	-1.46 (-3.30,0.37)	1.21 (-0.38,2.80)	-0.83 (-1.92,0.25)	-5.77 (-9.87,-1.67)
	RUCC3	-0.44 (-2.08,1.21)	1.68 (0.25,3.10)	-1.12 (-2.15,-0.08)	-7.53 (-10.95,-4.11)
	RUCC4	0.00 (-1.98,1.98)	2.44 (0.09,4.78)	-0.92 (-2.10,0.26)	0.74 (-5.01,6.49)
Dry continental	RUCC1	-0.78 (-2.51,0.96)	0.29 (-1.52,2.10)	-1.14 (-2.10,-0.17)	-1.67 (-6.04,2.69)
	RUCC2	1.76 (-0.29,3.81)	-0.75 (-2.95,1.46)	-0.73 (-1.81,0.35)	-0.05 (-5.61,5.51)
	RUCC3	0.57 (-0.91,2.05)	0.18 (-1.24,1.60)	-1.09 (-1.85,-0.32)	-1.77 (-4.38,0.84)
	RUCC4	0.33 (-1.08,1.73)	-0.35 (-2.01,1.31)	-0.11 (-1.08,0.86)	-2.00 (-6.33,2.34)
Hot summer	RUCC1	0.08 (-1.17,1.33)	-0.75 (-2.02,0.52)	0.13 (-0.86,1.12)	-0.32 (-3.76,3.12)

continental	RUCC2	0.36 (-1.36,2.09)	-1.15 (-2.63,0.33)	-0.45 (-1.47,0.58)	-1.39 (-5.73,2.95)
	RUCC3	0.69 (-0.40,1.78)	-1.02 (-2.15,0.11)	-0.25 (-1.06,0.55)	0.29 (-2.17,2.75)
	RUCC4	1.03 (-0.18,2.24)	0.22 (-0.94,1.39)	-0.05 (-0.96,0.85)	2.21 (-1.09,5.51)
Humid subtropical	RUCC1	-0.60 (-1.17,-0.03)	-0.77 (-1.25,-0.29)	-0.70 (-1.33,-0.07)	0.95 (-0.54,2.45)
	RUCC2	-0.22 (-1.23,0.79)	-1.40 (-2.26,-0.54)	-0.26 (-1.17,0.66)	1.34 (-1.06,3.73)
	RUCC3	-0.61 (-1.23,0.01)	-2.12 (-2.69,-1.55)	-0.50 (-1.12,0.11)	1.80 (0.33,3.28)
	RUCC4	-0.95 (-1.68,-0.23)	-1.96 (-2.66,-1.25)	-0.46 (-1.21,0.29)	1.89 (-0.03,3.81)
Mediterranean	RUCC1	-0.09 (-1.20,1.02)	0.37 (-1.05,1.80)	-0.41 (-1.37,0.55)	-1.57 (-4.84,1.70)
	RUCC2	0.66 (-1.04,2.36)	0.44 (-1.31,2.19)	0.12 (-0.95,1.19)	0.18 (-4.53,4.89)
	RUCC3	0.03 (-1.60,1.67)	0.71 (-1.02,2.44)	-0.32 (-1.33,0.69)	-2.77 (-7.18,1.65)
	RUCC4	0.83 (-1.08,2.73)	0.28 (-2.03,2.58)	-0.01 (-1.18,1.17)	0.65 (-4.67,5.98)
Warm summer continental	RUCC1	-0.10 (-1.18,0.98)	-1.29 (-2.35,-0.23)	0.06 (-0.77,0.88)	1.39 (-0.88,3.66)
	RUCC2	0.50 (-1.16,2.17)	-2.12 (-3.43,-0.82)	-0.19 (-1.17,0.79)	2.78 (-0.49,6.05)
	RUCC3	-0.40 (-1.63,0.84)	-2.37 (-3.44,-1.30)	-0.65 (-1.45,0.15)	0.11 (-2.16,2.38)
	RUCC4	-0.10 (-1.40,1.20)	-1.17 (-2.24,-0.10)	-0.10 (-0.97,0.77)	-0.63 (-3.32,2.05)
**Land**		**All-cause**	**Heart**	**Cancer**	**Stroke**
Dry	RUCC1	-6.16 (-10.20,-2.11)	8.92 (3.47,14.36)	7.73 (3.65,11.82)	11.35 (4.00,18.69)
	RUCC2	-0.63 (-5.60,4.34)	4.89 (-0.29,10.07)	4.29 (0.70,7.89)	10.57 (3.86,17.28)
	RUCC3	5.86 (2.39,9.33)	7.20 (2.26,12.14)	10.48 (7.11,13.86)	13.00 (7.32,18.68)
	RUCC4	4.17 (0.17,8.17)	8.80 (1.49,16.11)	4.94 (-0.60,10.47)	-1.13 (-11.43,9.17)
Dry continental	RUCC1	-0.94 (-4.11,2.23)	3.03 (-2.52,8.57)	3.89 (-0.40,8.19)	2.27 (-5.55,10.09)
	RUCC2	2.36 (-2.22,6.95)	0.18 (-6.23,6.58)	-0.97 (-6.58,4.65)	-0.12 (-9.97,9.74)
	RUCC3	1.62 (-1.41,4.65)	-0.81 (-5.04,3.42)	1.84 (-1.37,5.05)	0.50 (-4.85,5.84)
	RUCC4	4.00 (1.30,6.69)	1.50 (-3.21,6.22)	2.79 (-1.73,7.32)	4.84 (-2.98,12.66)
Hot summer continental	RUCC1	0.67 (-1.18,2.52)	-0.60 (-4.38,3.17)	0.37 (-2.23,2.98)	-1.45 (-6.98,4.08)
	RUCC2	-1.18 (-4.33,1.97)	-0.67 (-5.29,3.95)	-2.54 (-5.87,0.79)	2.90 (-4.78,10.59)
	RUCC3	-1.35 (-2.83,0.13)	-0.92 (-4.30,2.45)	-0.86 (-3.09,1.38)	-1.18 (-5.41,3.05)
	RUCC4	0.07 (-1.46,1.59)	0.76 (-2.61,4.13)	1.69 (-1.28,4.66)	-5.55 (-10.83,-0.26)
Humid subtropical	RUCC1	-0.65 (-1.28,-0.02)	-0.85 (-1.82,0.12)	0.09 (-0.48,0.66)	0.20 (-1.20,1.60)
	RUCC2	-0.03 (-1.25,1.18)	2.05 (0.03,4.08)	0.15 (-0.79,1.09)	1.44 (-1.41,4.28)
	RUCC3	-1.68 (-2.37,-0.99)	-1.20 (-2.28,-0.12)	-0.77 (-1.36,-0.18)	-1.78 (-3.31,-0.25)
	RUCC4	-1.06 (-2.01,-0.11)	-2.58 (-3.78,-1.38)	0.03 (-0.91,0.96)	-1.74 (-3.83,0.35)
Mediterranean	RUCC1	-2.20 (-5.16,0.76)	4.96 (0.10,9.82)	1.12 (-2.75,4.99)	2.97 (-3.36,9.31)
	RUCC2	0.20 (-4.53,4.93)	4.43 (-1.60,10.47)	-3.24 (-8.08,1.59)	0.40 (-8.53,9.33)
	RUCC3	-1.09 (-4.61,2.43)	4.38 (-1.29,10.04)	0.41 (-4.54,5.36)	4.54 (-3.89,12.97)
	RUCC4	-0.98 (-5.18,3.23)	1.28 (-5.81,8.36)	-2.04 (-8.15,4.08)	-0.83 (-10.44,8.78)
Warm summer continental	RUCC1	-0.66 (-2.90,1.57)	-2.38 (-5.53,0.77)	0.21 (-1.84,2.26)	-2.68 (-7.01,1.65)
	RUCC2	-0.51 (-3.81,2.79)	-4.45 (-8.34,-0.55)	1.28 (-1.59,4.15)	-4.83 (-10.97,1.31)
	RUCC3	-2.95 (-4.79,-1.11)	-5.33 (-7.94,-2.71)	-1.30 (-2.90,0.31)	-1.29 (-4.81,2.23)
	RUCC4	-3.77 (-5.71,-1.84)	-0.71 (-3.23,1.81)	-2.11 (-3.98,-0.24)	-1.58 (-5.78,2.63)
**Built**		**All-cause**	**Heart**	**Cancer**	**Stroke**

DOI: 10.1289/EHP119

Dry	RUCC1	5.11 (2.46,7.76)	1.05 (-2.80,4.90)	2.61 (-0.79,6.00)	5.05 (0.16,9.93)
	RUCC2	1.69 (-1.72,5.10)	-0.83 (-4.86,3.20)	1.21 (-2.97,5.39)	5.19 (0.20,10.19)
	RUCC3	-2.30 (-4.63,0.02)	-0.06 (-3.02,2.90)	-3.16 (-6.14,-0.18)	5.45 (1.56,9.35)
	RUCC4	-0.97 (-3.57,1.63)	0.01 (-3.45,3.47)	2.42 (-1.23,6.07)	0.16 (-5.66,5.99)
Dry continental	RUCC1	1.30 (-1.13,3.73)	-1.84 (-5.18,1.51)	2.39 (-0.64,5.42)	0.86 (-3.50,5.23)
	RUCC2	-1.47 (-4.56,1.62)	-1.60 (-4.88,1.67)	-0.78 (-4.26,2.69)	1.05 (-4.52,6.63)
	RUCC3	-1.54 (-3.50,0.42)	-4.46 (-6.58,-2.35)	-3.00 (-5.38,-0.62)	1.27 (-2.39,4.93)
	RUCC4	-1.46 (-3.13,0.21)	-1.20 (-3.49,1.08)	-0.84 (-3.02,1.34)	3.20 (-1.12,7.52)
Hot summer continental	RUCC1	-0.14 (-1.78,1.51)	-0.97 (-3.66,1.72)	-0.68 (-2.83,1.48)	-0.35 (-3.18,2.47)
	RUCC2	0.96 (-1.60,3.52)	-0.82 (-4.25,2.61)	1.19 (-2.46,4.83)	2.92 (-1.57,7.41)
	RUCC3	0.96 (-0.35,2.26)	-0.98 (-3.14,1.17)	1.54 (-0.34,3.42)	0.63 (-2.29,3.54)
	RUCC4	1.14 (0.06,2.22)	-1.60 (-3.49,0.30)	0.19 (-1.29,1.67)	-4.05 (-6.95,-1.14)
Humid subtropical	RUCC1	0.60 (-0.08,1.28)	-0.64 (-1.97,0.68)	-0.85 (-1.70,0.00)	0.13 (-1.06,1.32)
	RUCC2	0.60 (-0.70,1.91)	2.01 (-0.49,4.52)	0.88 (-1.73,3.49)	3.03 (-0.09,6.14)
	RUCC3	2.21 (1.47,2.95)	1.83 (0.63,3.03)	0.97 (-0.13,2.06)	3.50 (1.81,5.19)
	RUCC4	1.44 (0.71,2.16)	-0.54 (-1.83,0.75)	-0.11 (-1.08,0.87)	0.00 (-1.71,1.72)
Mediterranean	RUCC1	1.80 (-0.33,3.93)	-0.31 (-3.44,2.83)	1.50 (-1.66,4.66)	3.88 (0.97,6.80)
	RUCC2	0.19 (-2.97,3.35)	0.15 (-3.70,4.01)	1.16 (-2.81,5.12)	3.61 (-1.70,8.93)
	RUCC3	1.25 (-1.08,3.57)	-0.93 (-3.62,1.76)	1.94 (-1.25,5.13)	3.25 (-1.71,8.20)
	RUCC4	0.76 (-2.08,3.60)	-4.30 (-7.58,-1.01)	1.01 (-2.54,4.55)	0.59 (-4.87,6.06)
Warm summer continental	RUCC1	1.00 (-0.69,2.69)	-2.15 (-4.67,0.37)	2.32 (-0.05,4.70)	-2.44 (-4.89,0.02)
	RUCC2	0.25 (-2.09,2.60)	-3.18 (-6.41,0.06)	0.94 (-2.27,4.16)	-2.76 (-6.74,1.23)
	RUCC3	1.46 (0.08,2.85)	-3.16 (-5.44,-0.88)	-0.02 (-2.56,2.52)	0.73 (-2.47,3.93)
	RUCC4	3.44 (2.05,4.84)	-0.54 (-2.41,1.32)	3.46 (1.65,5.27)	-0.28 (-3.60,3.04)
**Sociodemographic**		**All-cause**	**Heart**	**Cancer**	**Stroke**
Dry	RUCC1	1.86 (-1.70,5.42)	2.54 (-2.13,7.22)	4.58 (1.66,7.51)	11.70 (6.46,16.93)
	RUCC2	2.77 (0.12,5.42)	2.78 (-1.24,6.79)	2.53 (0.34,4.71)	11.09 (6.42,15.76)
	RUCC3	3.84 (1.75,5.92)	4.24 (0.90,7.58)	4.24 (2.46,6.02)	10.11 (5.91,14.31)
	RUCC4	3.95 (0.36,7.55)	6.43 (0.17,12.70)	3.76 (0.97,6.55)	3.40 (-4.90,11.69)
Dry continental	RUCC1	0.43 (-1.73,2.59)	-0.96 (-4.25,2.33)	2.25 (-0.46,4.96)	1.52 (-3.90,6.94)
	RUCC2	-2.61 (-6.28,1.07)	-4.34 (-9.52,0.84)	-1.63 (-5.41,2.14)	1.43 (-5.35,8.21)
	RUCC3	-3.17 (-5.46,-0.88)	-1.51 (-4.74,1.72)	-0.22 (-2.15,1.71)	0.66 (-4.18,5.50)
	RUCC4	2.71 (0.05,5.37)	-2.97 (-6.47,0.54)	0.90 (-1.52,3.32)	8.52 (1.73,15.30)
Hot summer continental	RUCC1	-0.94 (-2.64,0.76)	-2.89 (-5.21,-0.57)	-0.30 (-2.05,1.44)	0.72 (-3.07,4.50)
	RUCC2	-2.30 (-5.26,0.66)	-4.78 (-8.53,-1.04)	-2.29 (-5.06,0.49)	4.14 (-1.23,9.51)
	RUCC3	-4.23 (-5.76,-2.70)	-3.95 (-6.64,-1.25)	-0.94 (-2.50,0.63)	0.94 (-2.46,4.35)
	RUCC4	0.25 (-1.42,1.91)	0.35 (-2.26,2.96)	2.03 (0.45,3.61)	1.99 (-2.36,6.34)
Humid subtropical	RUCC1	-0.30 (-0.98,0.38)	-1.76 (-2.90,-0.62)	0.32 (-0.27,0.91)	1.34 (-0.34,3.02)
	RUCC2	-1.01 (-2.58,0.56)	-6.21 (-8.50,-3.91)	0.51 (-0.88,1.89)	3.16 (0.41,5.90)
	RUCC3	-0.88 (-1.56,-0.21)	-4.66 (-6.23,-3.10)	0.31 (-0.49,1.11)	5.28 (3.52,7.05)
	RUCC4	-0.42 (-1.20,0.37)	-3.10 (-4.77,-1.43)	-2.07 (-3.05,-1.08)	7.68 (5.30,10.06)

DOI: 10.1289/EHP119

Mediterranean	RUCC1	-2.12 (-3.77,-0.46)	-2.76 (-5.57,0.05)	1.13 (-1.08,3.33)	2.54 (-1.16,6.23)
	RUCC2	-2.14 (-6.08,1.80)	0.72 (-3.95,5.40)	-1.79 (-5.37,1.79)	-0.54 (-6.88,5.80)
	RUCC3	-0.99 (-4.92,2.93)	1.00 (-3.48,5.49)	0.39 (-2.76,3.55)	4.97 (-1.22,11.15)
	RUCC4	-2.80 (-6.77,1.18)	-3.95 (-10.92,3.01)	-1.46 (-4.82,1.90)	2.92 (-4.72,10.56)
Warm summer continental	RUCC1	-0.90 (-2.07,0.27)	-5.00 (-6.91,-3.09)	0.52 (-0.95,2.00)	3.67 (0.42,6.91)
	RUCC2	-2.65 (-5.05,-0.25)	-9.00 (-12.61,-5.39)	0.30 (-2.20,2.79)	2.03 (-3.45,7.52)
	RUCC3	-2.76 (-4.70,-0.81)	-8.82 (-11.94,-5.71)	-2.00 (-3.74,-0.26)	3.28 (-1.00,7.56)
	RUCC4	0.41 (-1.56,2.38)	-4.34 (-7.49,-1.19)	0.61 (-0.82,2.04)	4.13 (-0.29,8.55)
